# Retrospective immunophenotypical evaluation of MET, PD-1/PD-L1, and mTOR pathways in primary tumors and pulmonary metastases of renal cell carcinoma: the RIVELATOR study addresses the issue of biomarkers heterogeneity

**DOI:** 10.37349/etat.2023.00165

**Published:** 2023-08-31

**Authors:** Melissa Bersanelli, Letizia Gnetti, Francesco Paolo Pilato, Elena Varotti, Federico Quaini, Nicoletta Campanini, Elena Rapacchi, Roberta Camisa, Paolo Carbognani, Enrico Maria Silini, Michele Rusca, Francesco Leonardi, Umberto Maestroni, Mimma Rizzo, Matteo Brunelli, Sebastiano Buti, Luca Ampollini

**Affiliations:** Université Côte d’Azur, France; ^1^Medical Oncology Unit, University Hospital of Parma, 43126 Parma, Italy; ^2^Pathologic Anatomy Unit, University Hospital of Parma, 43126 Parma, Italy; ^3^Pathologic Anatomy Unit, Azienda Socio-Sanitaria Territoriale di Cremona, 26100 Cremona, Italy; ^4^Medicine and Surgery Department, University of Parma, 43126 Parma, Italy; ^5^Thoracic Surgery Unit, University Hospital of Parma, 43126 Parma, Italy; ^6^Urology Unit, University Hospital of Parma, 43126 Parma, Italy; ^7^Medical Oncology Unit, Azienda Ospedaliero Universitaria Consorziale Policlinico, 70124 Bari, Italy; ^8^Pathologic Anatomy Unit, University Hospital of Verona, 37126 Verona, Italy; ^9^Medicine and Surgery Department, University of Verona, 37126 Verona, Italy

**Keywords:** Renal carcinoma, biomarkers, heterogeneity, immunohistochemistry

## Abstract

**Aim:**

In renal cell carcinoma (RCC), tumor heterogeneity generated challenges to biomarker development and therapeutic management, often becoming responsible for primary and acquired drug resistance. This study aimed to assess the inter-tumoral, intra-tumoral, and intra-lesional heterogeneity of known druggable targets in metastatic RCC (mRCC).

**Methods:**

The RIVELATOR study was a monocenter retrospective analysis of biological samples from 25 cases of primary RCC and their paired pulmonary metastases. The biomarkers analyzed included MET, mTOR, PD-1/PD-L1 pathways and the immune context.

**Results:**

High multi-level heterogeneity was demonstrated. MET was the most reliable biomarker, with the lowest intratumor heterogeneity: the positive mutual correlation between MET expression in primary tumors and their metastases had a significantly proportional intensity (*P* = 0.038). The intratumor heterogeneity grade was significantly higher for the mTOR pathway proteins. Combined immunophenotypical expression patterns and their correlations with the immune context were uncovered [i.e., mTOR expression in the metastases positively correlated with PD-L1 expression in tumor-infiltrating lymphocytes (TILs), *P* = 0.019; MET expression was related to PD-1 expression on TILs (*P* = 0.041, *ρ* = 0.41) and peritumoral lymphocytes (RILs; *P* = 0.013, *ρ* = 0.49)], suggesting the possibility of predicting drug response or resistance to tyrosine kinase, mTOR, or immune checkpoint inhibitors.

**Conclusions:**

In mRCC, multiple and multi-level assays of potentially predictive biomarkers are needed for their reliable translation into clinical practice. The easy-to-use immunohistochemical method of the present study allowed the identification of different combined expression patterns, providing cues for planning the management of systemic treatment combinations and sequences in an mRCC patient population. The quantitative heterogeneity of the investigated biomarkers suggests that multiple intralesional assays are needed to consider the assessment reliable for clinical considerations.

## Introduction

The treatment landscape of advanced or metastatic renal cell carcinoma (mRCC) has been widely renewed in the last few years. Two anti-programmed death protein-1 (PD-1) immune checkpoint inhibitors (ICIs), nivolumab and pembrolizumab, are now recommended as first-line therapy, each one associated with different tyrosine kinase inhibitors (TKIs) [1]. PD-1, the ICI target, belongs to the inhibitory B7-family molecules and is expressed by activated T-cells. Its primary ligand, programmed death-ligand-1 (PD-L1), is expressed by tumor cells, antigen-presenting cells (APCs), and non-hematopoietic stem cells. The PD-1/PD-L1 axis physiologically inhibits the effector T-cell activity in peripheral tissues during the inflammatory responses and in the limitation of autoimmunity. In the tumor microenvironment, the activation of this axis favors the immune escape of tumor cells. Most solid tumors have been demonstrated to express high levels of PD-L1; moreover, the relevance of PD-L1 expression on immune cells infiltrating the tumor also emerged, particularly on tumor-infiltrating lymphocytes (TILs). Nevertheless, these biomarkers’ prognostic and predictive role in mRCC is still unclear [2].

Furthermore, novel TKIs were included in these new strategies, extending the mechanism of action against new druggable targets beyond vascular endothelial growth factor receptors (VEGFRs). This is the case of cabozantinib, a small molecule TKI targeting mesenchymal-epithelial transition (MET) among other multiple kinases, now approved in combination with the anti-PD-1 nivolumab for the primary treatment of patients with mRCC [3]. Moreover, new multitarget TKIs are combined with older drugs, such as the mammalian target of the rapamycin (mTOR) inhibitor everolimus, now recommended in association with lenvatinib as a subsequent treatment line after progression to immunotherapy-based combinations [4–6].

Identifying reliable predictive biomarkers still needs to be met in this setting. Single-target attempts were performed in the case of ICIs, with the use of PD-L1 as likely prognostic or predictive of benefit, and several years before in the case of mTOR-inhibitors, with the identification of potentially predictive phosphorylated proteins in the mTOR-signal transduction pathway [7, 8].

Given the lack of trials directly comparing the several currently available ICI-based combinations, the choice between the different mechanisms of action is often aleatory, based on indirect comparisons from the literature and patients’ profiles and comorbidities. Identifying predictive molecular patterns of expression with combined biomarkers in mRCC would support the clinicians in choosing between the new combined treatment strategy, unveiling hypothesis-driven mechanisms of primary resistance even before initiating a treatment sequence [7, 8].

The main challenge of assessing biomarkers is represented by the natural tumor heterogeneity, intended and interpreted as a multilevel issue [9, 10]. If the term inter-tumoral heterogeneity identifies the heterogeneity between tumors (mRCC) of different individuals, the intra-tumoral heterogeneity can be defined as the heterogeneity between the primary renal cell carcinoma (RCC) and its metastases, and the intra-lesional heterogeneity is conceived as the heterogeneity of different sites within the same lesion (either the primary tumor or the metastasis).

With this complex issue in mind, the present study aimed to assess the currently used “druggable targets” and their molecular markers (i.e., phosphorylated proteins of their signal transduction pathways) with the accurate characterization of their inter-tumoral, intra-tumoral, and intra-lesional heterogeneity. The RIVELATOR study was designed to assess, by a reproducible, low-cost, and readily available laboratory technique, as immunohistochemistry (IHC), the expression of druggable targets of interest, including immune checkpoints (PD-1 and PD-L1) and proteins belonging to MET and mTOR pathways in mRCC. The explorative objectives of the analysis were the mutual correlations between clinical features and histopathological and immunophenotypical characteristics of the tumors.

Previously used methods to assess heterogeneity in this disease, i.e., in the case of multi-region sequencing, are limited by scarce availability in everyday practice, confining their use to purely scientific purposes [9]. One of the aims of this study was to provide an easy-to-use, reliable method to explore the issue in clinical practice.

A previous publication already described the immune context, expression patterns, and heterogeneity of the PD-1/PD-L1 immune checkpoint investigated in this study [11]. Herein, the findings about patterns of expression and heterogeneity concerning MET and mTOR pathways and their interplay with PD-1/PD-L1 expression status were reported, to identify and reveal systematic interactions between these druggable biomarkers, likely representing reproducible drug sensitivity patterns.

## Materials and methods

### Study design and inclusion criteria

The RIVELATOR study retrospectively enrolled consecutive patients who underwent surgical resection for synchronous or metachronous pulmonary metastases from mRCC in a single Thoracic Surgery Unit from 2000 to 2016 after resection of the primary tumor (previous partial or radical nephrectomy). The following inclusion criteria must be met: availability of both the primary tumor surgical samples and the resected pulmonary metastases in the Pathologic Anatomy Unit; availability of histologic material sufficient to perform all planned analyses on both sites; availability of clinical records at least until the time of metastasectomy.

### Study procedures

Clinical data collected from available records were: age at diagnosis/nephrectomy, gender, stage at diagnosis/nephrectomy, type of nephrectomy (radical or partial), the intent of nephrectomy (cytoreductive *vs.* curative); disease-free interval, defined as relapse-free survival (RFS) from nephrectomy and intended as the time from nephrectomy and the first histological or radiological assessment of disease recurrence; systemic intercurrent therapies for mRCC; time from nephrectomy to metastasectomy (defined as the surgical interval); type of pulmonary resection; time to the first recurrence after metastasectomy (defined as RFS from metastasectomy and intended as the time from metastasectomy and the first histological or radiological assessment of further disease recurrence); additional systemic treatments for mRCC after metastasectomy; updated survival status.

The study procedures, performed on both paired samples from the primary tumor and pulmonary metastasis, were:

(1) Complete histopathological revision, with the reassessment of histologic subtype, grading, size, (micro) vascular invasion, necrosis, and number of metastases.
(2) Immunohistochemical characterization, in terms of a qualitative and quantitative assessment of the subsequent biomarkers on tumor cells of both primary tumors and metastasis: PD-L1, MET (MET mutations were not assessed, the analysis only revealed the MET protein expression), phosphorylated-mTOR (hence called mTOR), phosphorylated eukaryotic translation initiation factor 4E binding protein 1 (4EBP1), hence called 4EBP1, and phosphorylated S6 ribosomal protein (S6RP), hence called S6RP.
(3) Qualitative and quantitative immunohistochemical assessment of PD-1 and PD-L1 on both TILs in the tumor context, defined as intratumoral lymphocytes (TILs), and in the peri-tumoral context, defined as peritumoral lymphocytes (RILs).

Tumor tissues were routinely formalin-fixed and paraffin-embedded (FFPE) and evaluated according to the recommendations of the World Health Organization (WHO) 2016 classification of tumors. Each FFPE was cut into histological sections for hematoxylin and eosin staining. Two expert pathologists (Letizia Gnetti and Francesco Paolo Pilato) revised all the original preparations for the study.

The most representative inclusion was selected for each case, and tissue sections were set up for the IHC analysis. IHC assays were performed according to standardized procedures. The immunoreactivity was evaluated with the antibodies listed in Table S1.

The complete analysis of qualitative and quantitative intralesional (within every single sample) and intratumoral (between primary tumor and its pulmonary metastasis) heterogeneity was also performed for PD-L1 with the following methods:

(1) The quantitative assessment of PD-L1 expression, in terms of the percentage of cells expressing the biomarker, was performed in each of the cases of the study population in four different high-power fields (HPFs) 40× magnification from separated samples of each district (tumor cells, TILs, and RILs) of the primary tumor and respective pulmonary metastasis.

(2) The qualitative assessment of PD-L1 expression, defined as the positivity for this biomarker on almost 1% of cells in each of the analyzed districts (tumor cells, TILs, and RILs) of the primary tumor and the respective pulmonary metastasis, was also performed.

(3) Four HPF 40× magnification from separated samples of each district (tumor cells, TILs, and RILs) of every single primary tumor and pulmonary metastasis were analyzed separately to compute the percentage of cells expressing PD-L1.

(4) Four HPF 40× magnification from separated samples of each district (tumor cells, TILs, and RILs) of every single pulmonary metastasis were analyzed separately for the percentage of cells expressing PD-L1.

(5) Immunophenotypical results from the IHC analysis of MET, mTOR, 4EBP1, S6RP, PD-1, and PD-L1 were quantitatively and qualitatively compared between each primary tumor and its metastasis, assessing intratumoral heterogeneity.

The investigated biomarkers’ immunohistochemical expression was evaluated using a semi-quantitative optical microscope and independently reviewed by two experts (Letizia Gnetti and Francesco Paolo Pilato). Discordant cases, or cases with no homogeneous staining due to necrotic areas, were revised in consensus. Samples lacking valid internal control were excluded, or the IHC procedure was repeated on new sections from the original tissue block.

For each biomarker, the qualitative assessment provided the immunohistochemical finding of positivity or negativity according to the validated criteria from the literature. The quantitative assessment was performed with different methods, respectively used (each of them) for each biomarker:

(1) Absolute percentage of positive cells.

(2) Thresholds of 1%, 10%, 50%, and 70% of positive cells.

(3) Expression intensity from 0 to 1+, 2++, 3+++.

(4) Intensity score of 0 (including 0 and 1+) or 1 (including 2++ and 3+++).

Each of the parameters assessed in the study was descriptively and statistically analyzed to investigate any mutual correlation or relationship between clinical features, histopathological characteristics, and immunophenotypical elements.

### Descriptive and inferential statistics

Respectively, a descriptive analysis of data and inferential statistical analysis were performed according to the following methods. To study the correlation between the various parameters, the *t-*test was used for the parametric data and Pearson’s *χ*^2^ test or the *Fisher* test for non-parametric data. Non-parametric statistics with Kaplan-Meier curves were used to estimate the survival function. Survival times analyzed with descriptive aim included: RFS from nephrectomy, RFS from metastasectomy, surgical interval, and overall survival (OS) from nephrectomy and metastasectomy, respectively. OS was measured as the time between the date of the primary surgical intervention and the date of death for any reason or the last follow-up for live patients.

## Results

### Study population

Overall, 25 patients were retrospectively enrolled in the RIVELATOR study. They underwent radical or partial nephrectomy from September 1998 to October 2014 and underwent surgical resection of lung metastases between 2000 and 2016. The clinical characteristics of the study population were reported in Table S2.

The median follow-up from renal cancer diagnosis was 9.8 years, and the median follow-up from metastasectomy was 7.1 years. No patients were lost at follow-up. For all patients, nephrectomy coincided with the renal cancer diagnosis because no biopsies (or metastasectomy) with diagnostic intent were performed in any patient before the surgical procedure. After nephrectomy, the first distant tumor relapse was surgically treated in all 21 patients with primary early-stage disease, preceded or not by an active surveillance period. The metastasectomy immediately followed the prior nephrectomy in the other 4 cases, with synchronous metastases.

At the time of the pulmonary metastasectomy, five patients had received systemic treatment for renal cancer in the adjuvant setting (3 cases, respectively, with cytokines or sunitinib in clinical trials) or as first-line treatment for metastatic disease (2 patients, both with sunitinib), according to the contemporary clinical practice.

Clinical data about the follow-up after pulmonary resection were available for 16 patients out of 25, 5 did not have a relapse, while eleven experienced recurrences of disease during the follow-up period, ten of which had thoracic recurrence (lung or lymph node metastasis).

Clinical data about possible systemic treatments after metastasectomy were available for 15 patients, of which five were not treated (four free from disease and one unfit for therapy despite metastatic), and ten received oral TKIs as first-line treatment (50% pazopanib and 50% sunitinib), according to contemporary clinical practice.

### Clinical outcome of patients

At the median follow-up of 9.8 years [95% confidence intervals (CI) = 8.7–10.9], the median OS (mOS) of the study population from renal cancer diagnosis, as to say from nephrectomy, was of 8.1 years (95% CI = 3.7–12.5), with 13 (52%) censored cases (Figure S1). The estimated survival was 96% at one year, 88% at 3 years, 74% at 5 years, of 48% at 10 years.

The mOS from pulmonary metastasectomy (Figure S2) was of 5.5 years (95% CI = 1.9–9.1), with 13 censored cases (52%) at the median follow-up of 7.1 years from lung resection. The estimated survival was 88% at one year, 64% at 3 years, 58% at 5 years, and 43% at 10 years.

The median time between nephrectomy and metastasectomy was 32.6 months (95% CI = 15.7–49.4, no censored cases; mean of 35.4 months). The median time to the first disease relapse (radiologically or histologically assessed) after nephrectomy, defined as RFS from RCC diagnosis, which was evaluable for the 21 patients with the metachronous occurrence of metastases (not applicable for the 4 cases with advanced disease at the time of nephrectomy), was of 25.9 months (Figure S3), widely ranging from 1.3 months to 96.9 months.

The first relapse of disease after nephrectomy coincided with the pulmonary metastases resection in only 3 over 21 patients. Three patients had a prior surgical intervention before the metastasectomy considered for our study (due to adrenal or pulmonary relapse). For the remaining 15 patients, the first relapse coincided with the radiological finding of the subsequently resected pulmonary metastases. Still, the surgical intervention was delayed after systemic therapy or active disease surveillance, ranging from 1 month to 30 months. The median time from the first relapse of the disease to the pulmonary metastasectomy was 6.1 months.

At 24 months from nephrectomy, 13 patients were free from recurrence, with a 2-year RFS of 61.9%; at 36 months, the RFS was 38% (8 patients free from a relapse); the 5-year RFS was 9.5% (2 cases without recurrence at 60 months from nephrectomy).

Finally, only 16 patients were evaluable for RFS after pulmonary resection of metastases (data not available for 9 patients): their median RFS was 52.9 months (95% CI = 0–163.2; 5 censored cases); their RFS at 6 months was 68.8% (all patients still evaluable, 11 of them free from relapse). The estimated 2-year RFS was 56.3%, and at 5 years, it was 45% (Figure S4).

### Histopathological features

**Figure 1:**
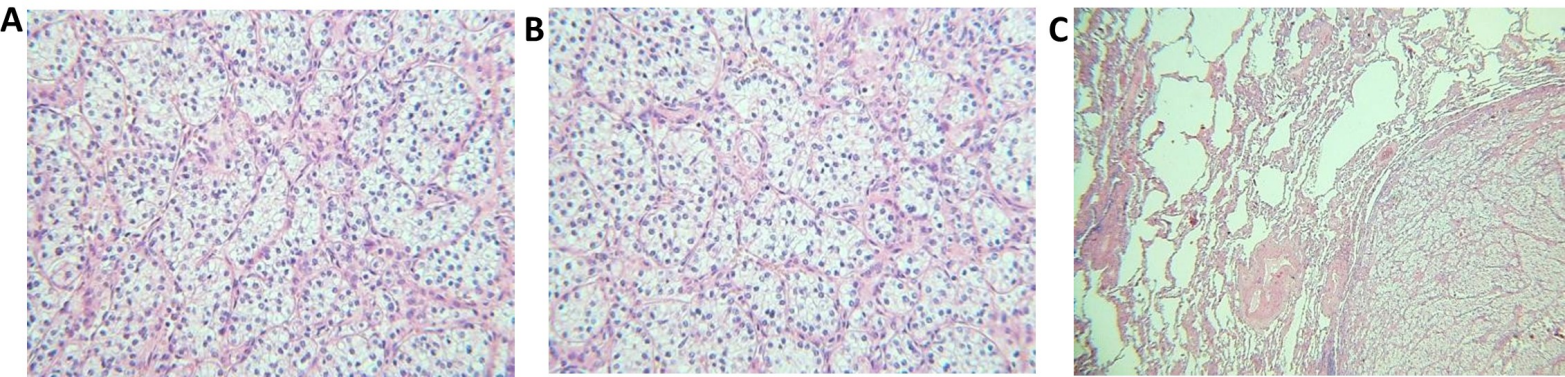
Histopathological images of the tumors. (A) Clear-cell RCC, primary tumor (40× magnification); (B) clear-cell RCC, primary tumor (40× magnification); (C) pulmonary metastasis of RCC (20×, magnification)

The primary tumor’s histologic subtype was clear-cell RCC (ccRCC) for 24 patients; only one patient had mixed histology with clear cell and papillary components. Histopathological images of samples collected for the histology revision are shown in Figure 1.

The International Society of Urological Pathology (ISUP) grade was G2 for 5 cases, G3 for 14 cases, and G4 for 6 cases (no G1 cases); fifteen tumors presented vascular invasion; the presence of necrosis was 0 in 9 cases, 1+ in 5 cases, 2++ in 7 cases, 3+++ in 4 cases. The other histopathological characteristics of interest are reported in Tables 1 and 2.

**Table 1:** Histopathological features of the analyzed samples from nephrectomies

**Variables**	**Primary tumor**	**Number of patients (%)**
T size	≤ 4 cm	3 (12)
	> 4 cm but ≤ 7 cm	12 (48)
	> 7 cm but ≤ 10 cm	6 (24)
	> 10 cm	4 (16)
Histotype	Clear cell	24 (96)
	Sarcomatoid	0 (0)
	Others	1 (4)
ISUP grade	G1	0 (0)
	G2	5 (20)
	G3	14 (56)
	G4	6 (24)
Vascular invasion	Yes	15 (60)
	No	10 (40)
Necrosis	0	9 (36)
	1 (+)	5 (20)
	2 (++)	7 (28)
	3 (+++)	4 (16)

**Table 2:** Histopathological features of the analyzed samples from pulmonary resections

**Variables**	**Pulmonary metastasis**	**Number of patients (%)**
Number of metastases	Single	12 (48)
	Multiple	13 (52)
Histotype	Clear cell	23 (92)
	Sarcomatoid	1 (4)
	Others	1 (4)
Laterality	Left lung	7 (28)
	Right lung	14 (56)
	Bilateral involvement	4 (16)
Nodal involvement	Yes	3 (12)
	No	3 (12)
	Unknown (not resected)	21 (84)
Necrosis	0	17 (68)
	1 (+)	3 (12)
	2 (++)	2 (8)
	3 (+++)	3 (12)

The histology of metastases and primary tumors matched in 24 cases while differed in one last ccRCC case displaying dedifferentiated metastatic sarcomatoid RCC. The presence of necrosis in the metastases was lower than that in primary tumors: on average, it was unchanged between primary and secondary lesions in 32% of cases, decreased in 48%, and increased in 20% (see Tables 1 and 2).

### Tumor immunophenotype

From the evaluation of the 25 paired samples of primary renal cancers and their respective pulmonary metastases, detailed immunophenotypical data were obtained about the qualitative and quantitative expression of the subsequent biomarkers:

(1) MET on tumor cells.

(2) Phosphorylated forms of mTOR, 4EBP1, and S6RP on tumor cells.

MET was expressed by more than 90% of both primary (T) and metastatic (M) lesions, often with high intensity (especially in metastases).

mTOR and 4EBP1 were frequently expressed, with a range of 76–80% of cases for the first and reaching 100% of cases in the metastases for the latter, which mean intensity was also notable. In comparison, S6RP had a relatively lower expression in the primary tumor (24%), notwithstanding high expression in the metastases (72%).

**Figure 2:**
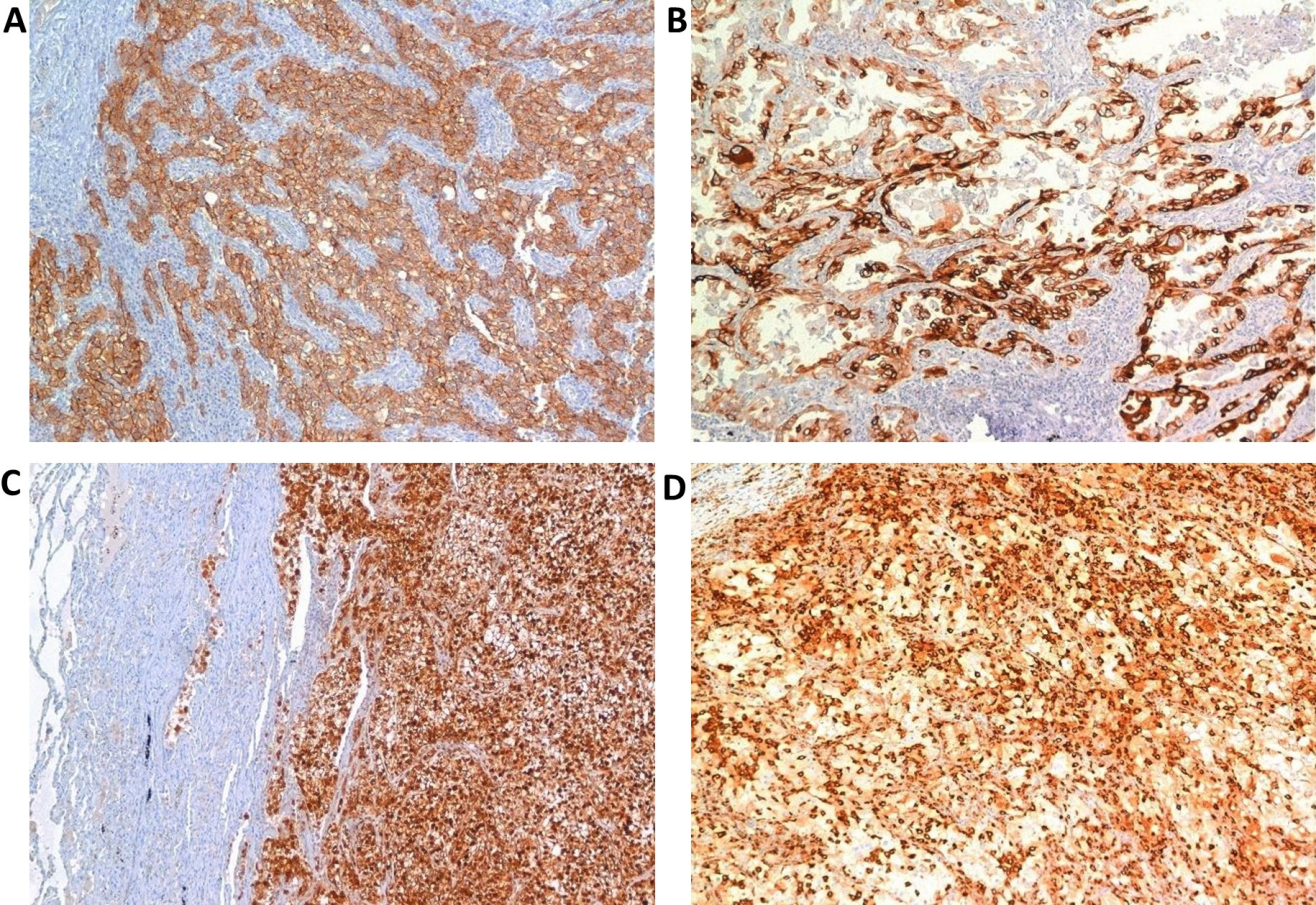
Positive IHC assay in primary RCC (40× magnification). (A) MCT; (B) phosphorylated-mTOR; (C) phosphorylated-EBP1; (D) phosphorylated-S6RP

Qualitative assessments of all these biomarkers are reported in Table 3, while quantitative data are summarized in Table 4. Images of the immunohistochemical detection of each biomarker are shown in Figure 2.

**Table 3:** Qualitative results of the analyzed biomarkers in the study population

**Biomarker**		**Primary tumor**		**Pulmonary metastasis**	
		**Number of cases**	**%**	**Number of cases**	**%**
MET	+	23	92	24	96
	-	2	8	1	4
mTOR	+	20	80	19	76
	-	5	20	6	24
4EBP1	+	22	88	25	100
	-	3	12	0	0
S6RP	+	6	24	18	72
	-	19	76	7	28

+: positive; -: negative

**Table 4:** Quantitative results of the analyzed biomarkers in the study population

**Biomarker**		**Primary tumor**	**Metastasis**
MET (% positive cells)	Mean	44.6	72.4
	Median	50	90
	Range	0–100	0–100
MET (number of cases)	Negative	2	1
	1 (+)	12	3
	2 (++)	7	7
	3 (+++)	4	14
mTOR (% positive cells)	Mean	28.1	26.4
	Median	30	10
	Range	0–80	0–90
mTOR (number of cases)	Negative	5	6
	1 (+)	2	12
	2 (++)	6	2
	3 (+++)	12	5
4EBP1 (% positive cells)	Mean	42.8	82.8
	Median	30	100
	Range	0–100	5–100
4EBP1 (number of cases)	Negative	3	0
	1 (+)	8	2
	2 (++)	6	5
	3 (+++)	8	18
S6RP (% positive cells)	Mean	5.8	32
	Median	0	20
	Range	0–70	0–100
S6RP (number of cases)	Negative	19	5
	1 (+)	3	8
	2 (++)	3	5
	3 (+++)	0	6

### Tumor heterogeneity

The intratumoral heterogeneity analysis, from the primary tumor to its pulmonary metastasis and intralesional heterogeneity (in each sample), revealed informative results regarding clinically meaningful histopathological characteristics, the immune context, and single biomarkers, as described below. Data about each biomarker’s intratumoral heterogeneity are summarized in Tables S3 and S4, reporting the different expression levels between primary tumors and pulmonary metastases.

### Biomarker correlations

The following subparagraphs report all the statistically significant and potentially clinically meaningful mutual correlations between druggable biomarkers of interest and, in turn, all the biomarker’s correlations with clinical and histopathological variables.

#### MET expression

The quantitative expression of MET on primary tumors (T) was positively related to that measured at metastatic sites (M, *P* = 0.029, *ρ* = 0.44). T MET and M MET values were also associated with the following parameters:

(1) PD-1 expression intensity on TILs (*P* = 0.041, *ρ* = 0.41) and RILs (*P* = 0.013, *ρ* = 0.49) of the tumor, respectively.
(2) The intensity of tumor 4EBP1 expression (*P* = 0.03, *ρ* = 0.43).
(3) Higher grading (*P* = 0.011, *ρ* = 0.5).
(4) The positive mutual correlation between T MET and M MET quantity had a significantly proportional intensity (*P* = 0.038); primary tumors with MET expression > 10% had a high probability of having MET expression over 70% on pulmonary metastases [odd ratio (OR) = 26.7, 95% CI = 2.2–317.2, *P* = 0.006].

MET expression intensity at metastatic sites was positively related to the following:

(1) The intensity of expression of 4EBP1 on the metastasis (*P* = 0.008, *ρ* = 0.51).
(2) Tumor grading: metastases with higher MET expression belonged to primary tumors of higher grade (*P* = 0.011, *ρ* = 0.5).

#### mTOR pathway proteins

Primary tumors with high expression of mTOR (> 50%) had a higher probability and intensity of expression of 4EBP1 (*P* = 0.027) and S6RP (*P* = 0.017). Similarly, pulmonary metastases with high mTOR expression (> 50%) had a higher probability and intensity of S6RP expression (*P* = 0.035).

Positive mTOR immunolabelling on primary tumors was positively related to the probability of mTOR expression > 10% on pulmonary metastases (*P* = 0.046).

In addition, the extent of mTOR expression on primary tumors was proportional to PD-L1 expression intensity in TILs located at metastatic sites (*P* = 0.001, *ρ* = 0.6).

It was more likely for mTOR-positive tumors to have moderate or intense amounts (correspondent to score 1) of TILs (*P* = 0.046), and in cases with mTOR expression > 50%, a significant increase in RILs was observed (*P* = 0.036).

4EBP1 expression levels on the primary tumor resulted positively related to MET expression (see sub-paragraph “MET expression”); such correlation was robust (OR = 6.75, 95% CI = 1.2–39.2, *P* = 0.047) when considering expression thresholds higher than 50%.

S6RP expression intensity on the primary tumor was positively related to the number of RILs (*P* = 0.038) and positively correlated with PD-L1 intensity on RILs of pulmonary metastases (*P* = 0.018, *ρ* = 0.47).

mTOR expression at metastatic sites was inversely related to the presence of necrosis (*P* = 0.018); conversely, tumors with more necrotic metastases were less likely to express mTOR *in situ* and on pulmonary metastases (*P* = 0.029), thus excluding an assessment bias caused by the presence necrosis.

At metastatic sites, the expression of mTOR positively correlated with PD-L1 expression in TILs (*P* = 0.019, *ρ* = 0.47), and 4EBP1 expression was positively related to MET with proportional intensity levels (*P* = 0.001). For respective expression thresholds higher than 50% of cells (OR = 28.5, 95% CI = 1.9–420.6, *P* = 0.016). Moreover, 4EBP1 expression on pulmonary metastases tended to proportionally increase with the size of primary tumors (*P* = 0.003, *ρ* = 0.57).

## Discussion

The RIVELATOR study results demonstrated the inter-tumoral, intra-tumoral, and intra-lesional heterogeneity of crucial druggable targets potentially exploitable as predictive biomarkers for systemic treatment in mRCC. The first deduction that can be drawn from these results is that such multi-level heterogeneity is likely responsible for both primary and acquired drug resistance due to dissociated responses and progressive clone selection. At the same time, the multi-level assessments used in the present study allowed the identification of different combined expression patterns in the analyzed cases, providing cues for planning the management of systemic treatment combinations and sequences in an mRCC patient population.

MET seems the most reliable among the investigated biomarkers and has the lowest intratumor heterogeneity, at least in terms of qualitative concordance between the primary tumor and its metastasis. The literature already reported its expression in RCC as high (80% of cases in old retrospective studies on primary tumors), and such a finding was confirmed in our RIVELATOR population [12, 13]. The added value of its assessment in paired secondary lesions documented here confirms the potential of this pathway as an ideal therapeutic target in mRCC.

MET association with PD-1 expression in TILs and RILs suggests that T-cell exhaustion could be relevant in MET-positive mRCC, encouraging combining a MET-directed TKI with an ICI for treating tumors carrying this immunophenotypic pattern. Considering both the biological aggressiveness (high grade) and the frequently intense expression of 4EBP1 in these same tumors, the subsequent therapeutic strategy could rely on an mTOR-inhibitor-based combination as a preferential second-line choice (i.e., lenvatinib plus everolimus).

The grade of intratumor heterogeneity was relatively high for the mTOR pathway, as 60% of cases varied for at least one protein (mostly S6RP) from a qualitative point of view, primarily due to S6RP. S6RP was the most frequently affected by intratumor variability among the different biomarkers. Such results suggest that mTOR could be more likely the main driver only for sub-clonal cell populations in RCC, alternatively increasing or decreasing within the overall tumor volume according to the time and the selective pressure of systemic therapies. Nevertheless, the mTOR pathway remains of potential relevance as a therapeutic target. However, its natural alternating prevalence could represent a plausible explanation for a dissociated disease response in the case of monotherapy. Based on this contention, combination therapies with different mechanisms of action, such as everolimus plus lenvatinib, could allow better therapeutic management of such jeopardized diseases.

Prior retrospective studies analyzed the prognostic and predictive role of biomarkers related to the mTOR pathway, assessed only on the primary renal cancer, underlining the poor prognostic role of 4EBP1 expression, which seemed to be related to high-grade and stage of tumors and to worse RFS, as well as S6RP expression [14, 15]. Of interest, previous data suggested the potentially powerful prognostic and predictive value of S6RP and mTOR for the efficacy of everolimus monotherapy in advanced RCC patients [16].

In the present case series, an mTOR-activated pattern associated with PD-L1 expression in RILs was identified. Considering the unconfirmed predictive role of PD-L1 expression in mRCC treated with ICIs, reliable predictive considerations cannot be drawn. Nevertheless, the documented correlations between the expression of the phosphorylated proteins of the mTOR pathway and the presence of more significant amounts of TILs and RILs suggest that mTOR-driven tumors are more immunogenic. Another valuable piece of information with a clinical readout that emerged from the study is represented by the lack of an mTOR-positive pattern in mRCC with necrotic metastases, possibly discouraging the use of mTOR inhibitors (especially as a single agent) in these cases.

Considering the current availability of multiple second-line treatment choices for mRCC [1, 6], without reliable comparative evidence among different and exploitable mechanisms of action, the present investigation included the aim of identifying tumors in which the mTOR axis constitutes a relevant druggable driver of the disease (or at least the driver of prevalent sub-clones). These results (Tables S3 and S4) demonstrated that the expression of the three activated mTOR-related proteins was not casual or independent. Still, instead, they were all mutually correlated, fostering the hypothesis of a pivotal role of such a pathway, at least in a subgroup of mRCC cases. The mutual relationship remained true also in the pulmonary metastasis; furthermore, mTOR expression resulted in being homogeneous between paired primary and secondary lesions, confirming its role in disease evolution. The quantitative heterogeneity of all the investigated biomarkers strongly suggests that multiple intralesional assays are needed to consider the assessment reliable for such clinical considerations.

The main limitations of the RIVELATOR study are the relatively limited sample size and the wide temporal distribution of cases over time. These elements are both due to the unusual setting’s peculiarity, represented by candidates for radical surgery after metastatic relapse in the pulmonary district. This clinical homogeneity was required to avoid gross biases when investigating different molecular pathways. Due to the limited sample, inferential statistics were only feasible for some of the variables considered in this study. Another limitation is that unavoidable clinical biases prevent the assessment of any conclusive prognostic or predictive value of the analyzed biomarkers. Only a minority of patients included in this study were treated with systemic therapies, and, at the time of the RIVELATOR study conduction, cabozantinib (the only drug targeting MET currently used in renal cancer) and nivolumab (targeting PD-1) were not yet approved. The present study population, treated with old-generation compounds or not receiving systemic therapies, should be considered a development setting, allowing the identification of proper methods and recognition of reliable immunophenotypical patterns. Notwithstanding, the aim of the study was entirely descriptive and focused on non-clinical endpoints, allowing the identification and characterization of intratumoral and intralesional heterogeneity of mRCC concerning druggable biomarkers of interest. Another undeniable limitation is the multiplicity bias concerning the mutual correlations evidenced between the variables included. Despite these limitations, findings of this study provide accurate information that can contribute to the clinicians’ awareness concerning the molecular heterogeneity of mRCC, discouraging therapeutic choices based on single biomarkers, mainly when assessed on a single sample or site of disease.

The main conclusion of the present study is the warning about the reliability of the identification of immunophenotypical patterns of mRCC, which is heavily affected by procedural issues when heterogeneity is not adequately considered. When using immunophenotypic biomarkers with prognostic or predictive purposes, especially to investigate intrinsic resistance of the disease to systemic treatments, we strongly suggest a multi-level assessment with paired analyses on both the primary tumor and a representative metastasis and with multiple assays within the same lesion. The results of the RIVELATOR study demonstrate that a single assay on a unique biopsy would not be reliable for predictive purposes toward systemic therapy for mRCC.

Further development of the RIVELATOR study involves the validation of the methods and the identified molecular patterns on a prospective population of mRCC patients undergoing sequential lines of treatment with ICI-based, MET-based, or mTOR-based drug combinations to test their true predictive potential toward systemic treatment resistance or sensitivity and clinical outcome following these new treatment opportunities.

## Abbreviations

CI: confidence interval
HPFs: high-power fields
ICIs: immune checkpoint inhibitors
IHC: immunohistochemistry
MET: mesenchymal-epithelial transition
mRCC: metastatic renal cell carcinoma
mTOR: mammalian target of the rapamycin
PD-1: programmed death protein-1
PD-L1: programmed death-ligand-1
RCC: renal cell carcinoma
RFS: relapse-free survival
RILs: peritumoral lymphocytes
RIVELATOR: retrospective immunophenotypical evaluation of MET, PD-1/PD-L1, and mTOR
S6RP: S6 ribosomal protein
TILs: tumor-infiltrating lymphocytes
TKIs: tyrosine kinase inhibitors
4EBP1: eukaryotic translation initiation factor 4E binding protein 1

## Supplementary materials

The supplementary material for this article is available at: https://www.explorationpub.com/uploads/Article/file/1002165_sup_1.pdf.

## Declarations

### Availability of data and materials

All datasets for this study are included in the manuscript and the supplementary files.

### Copyright

© The Author(s) 2023.
