# Supplementary material for: Retrospective immunophenotypical evaluation of MET, PD-1/PD-L1, and mTOR pathways in primary tumors and pulmonary metastases of renal cell carcinoma: the RIVELATOR study addresses the issue of biomarkers heterogeneity
Source: Explor Target Antitumor Ther. 2023 Aug 31;4(4):743–56. doi: 10.37349/etat.2023.00165 (PMC10501858; doi:10.37349/etat.2023.00165)
Supplement: Supplementary file 1 [file 1002165_sup_1.pdf]

Table S1. List of the antibodies used for the immunohistochemical assay

Antibody	Clone	Company	Dilution
PD-1	NAT105	Roche	Ready to use
PD-L1	22C3	Dako	1:50
c-Met	SP44	Roche	Ready to use
Phospho-4e-BP1	Thr37/46	Cell Signaling	1:50
Phospho-S6R	Ser235/236	Cell Signaling	1:50
Phospho-m-Tor	Ser2448	Cell Signaling	1:50

Table S2. Clinical characteristics of the study population

Clinical variables	N° of patients	% of patients*
**Gender**		
M	15	60%
F	10	40%
**Stage at diagnosis**		
I	11	44%
II	6	24%
III	4	16%
IV	4	16%
**Type of nephrectomy**		
Partial	1	4%
Radical	24	96%
with lymphadenectomy	1	4%
with adrenalectomy	4	16%

**Intent of nephrectomy**		
palliative (cytoreduction)	4	16%
curative	21	84%
**Systemic treatment after nephrectomy**		
Any	20	80%
Adjuvant	3	12%
I line	2	8%
**Type of pulmonary surgery**		
metastasectomy	11	44%
wedge resection	9	36%
lobectomy	4	16%
bilobectomy	1	4%
with lymphadenectomy	6	24%
**Relapse after pulmonary surgery**		
Yes	11	44%
No	5	20%
Unknown	9	36%
**Systemic therapy after relapse**		
Yes	10	40%
No	5	20%
Unknown	10	40%

* The percentage was evaluated based on the total number of patients which were evaluable for the parameter.

** The median age of patients was 65 years (range 55–78)

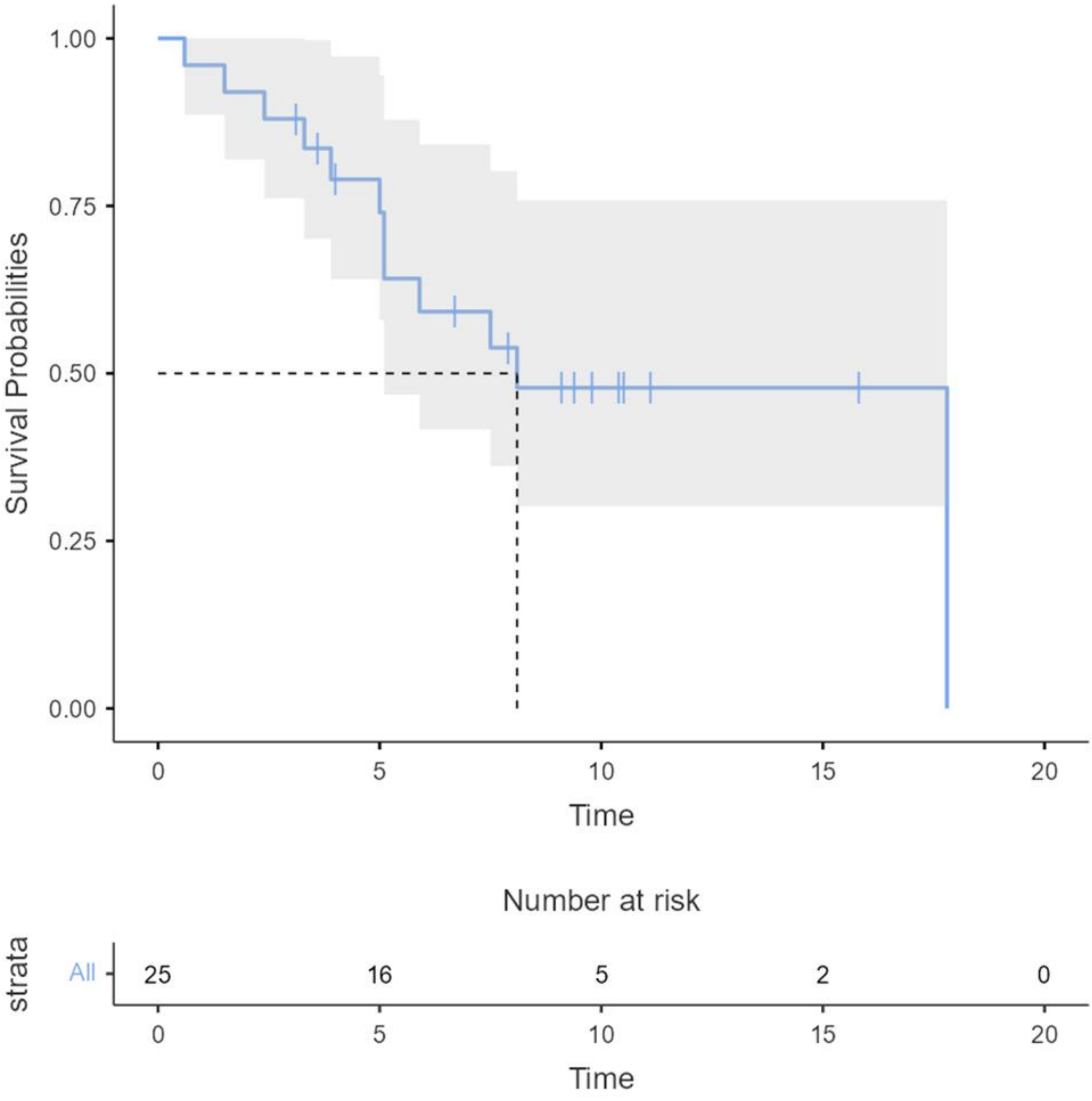

Figure S1. Overall survival of the study population from the first diagnosis of renal cancer (coincident with the time of nephrectomy in all cases)

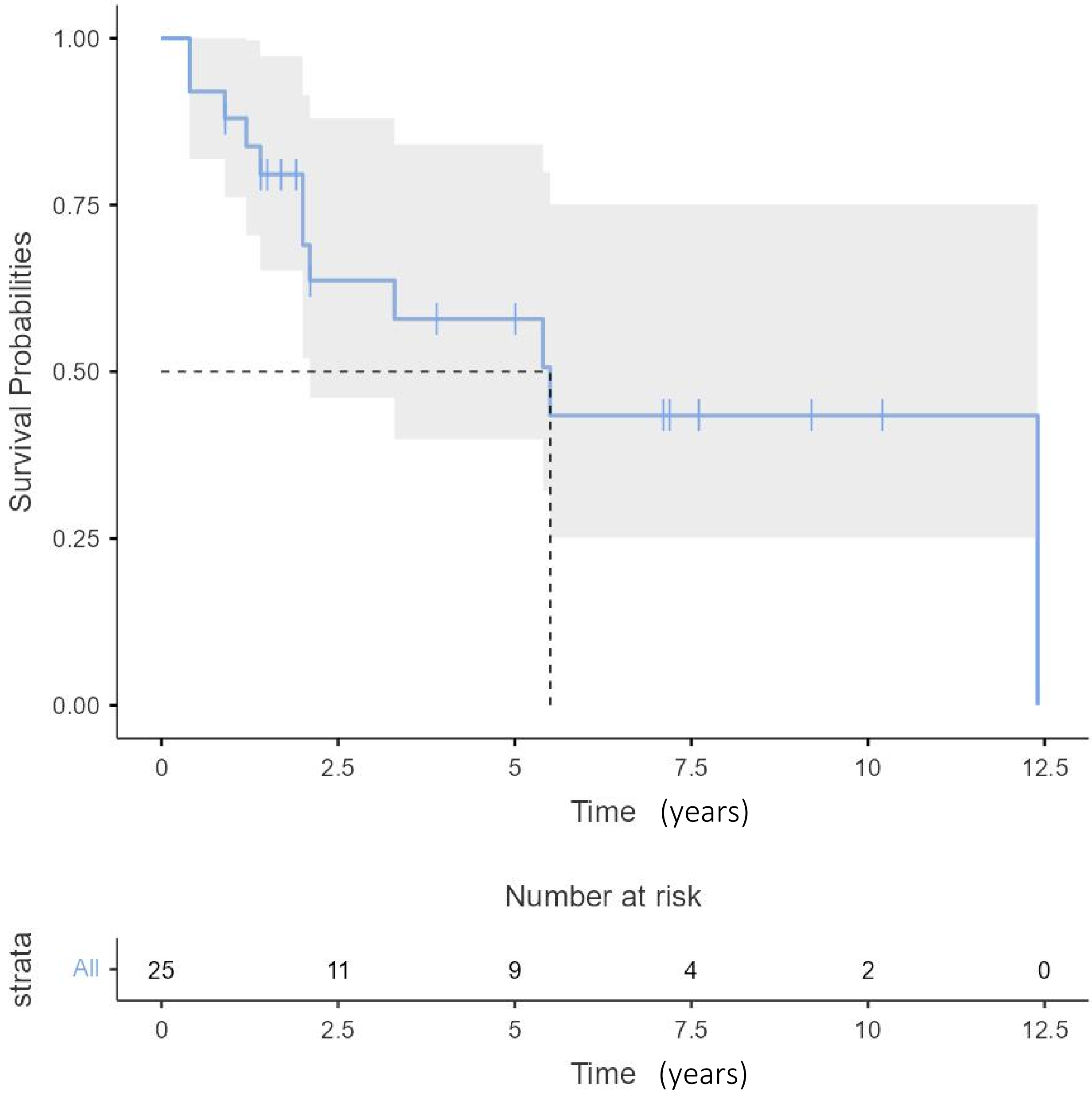

Figure S2. Overall survival of the study population from pulmonary metastasectomy

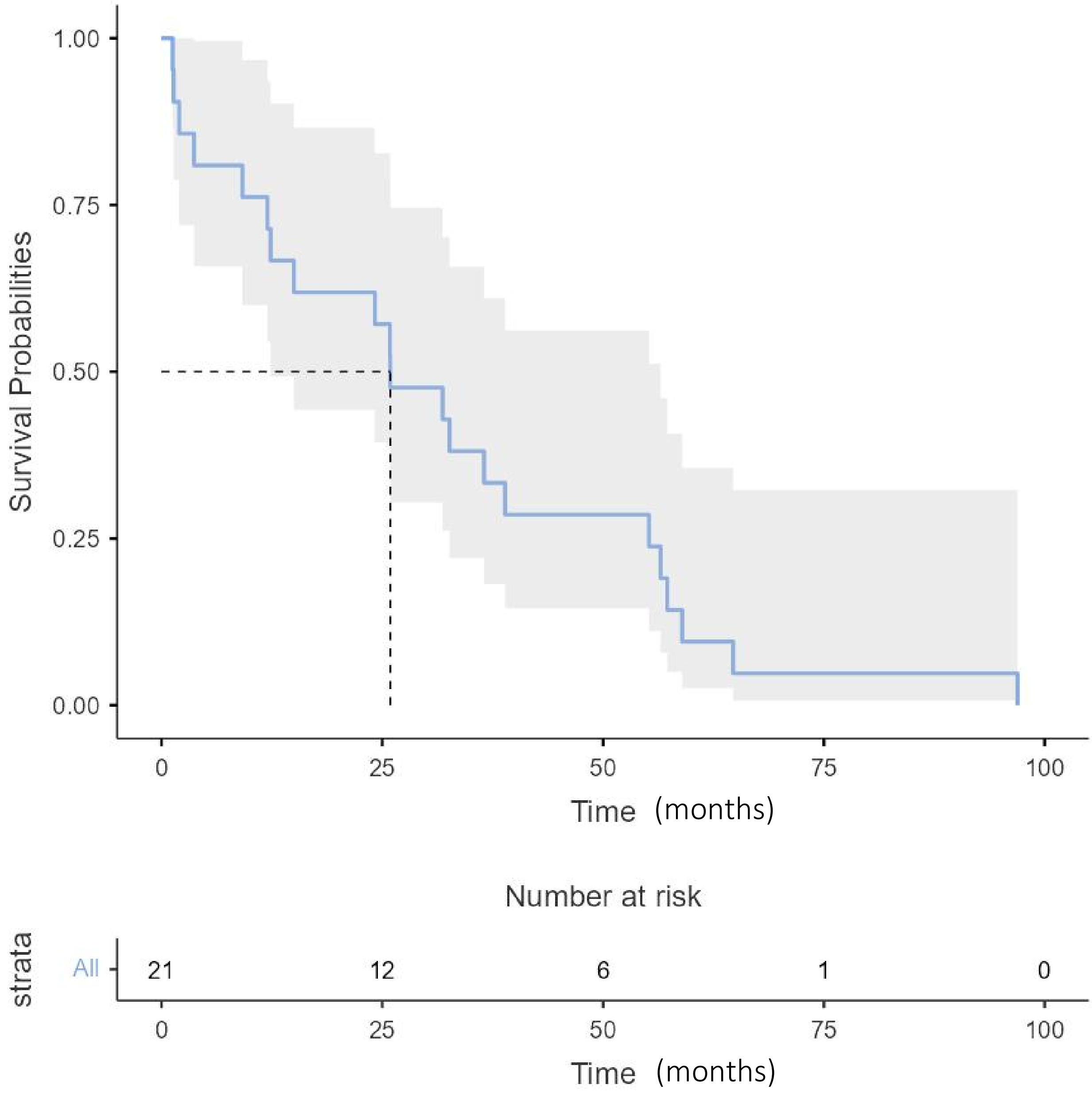

Figure S3. Relapse-free survival from nephrectomy in the overall population

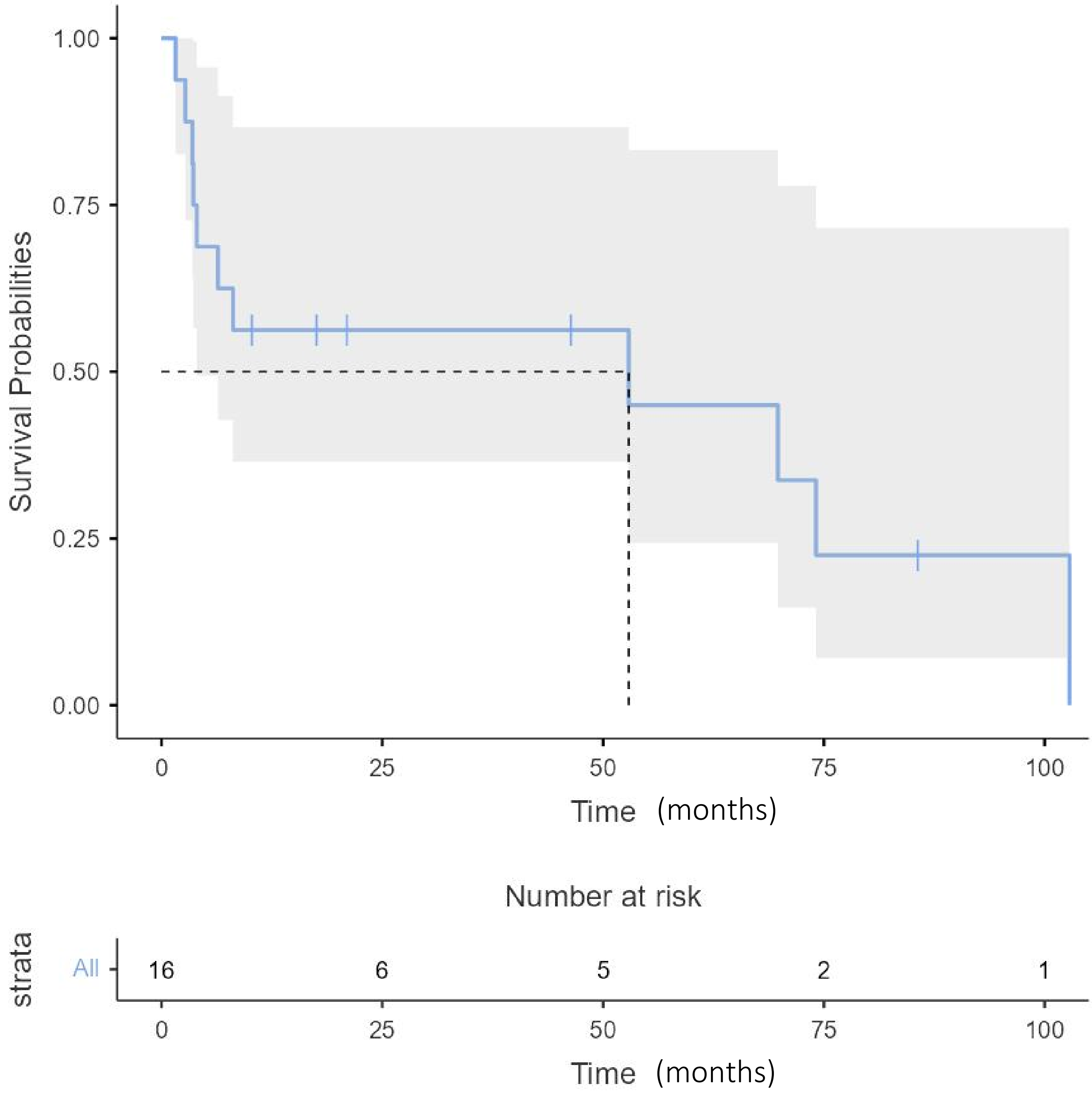

Figure S4. Relapse-free survival from pulmonary metastasectomy in the evaluable population

Table S3. Intratumor heterogeneity (ITH) between qualitative expression of biomarkers belonging to MET and mTOR pathways in the primary tumor (T) and the respective pulmonary metastasis (M). The ITH grade was low for the MET pathway (only one case with a heterogeneous expression) but instead relatively high for the mTOR pathway (15 cases of ITH). Among the different biomarkers, S6RP was the most frequently heterogeneous (14 discordant cases). Heterogeneous cases (for at least one parameter) are reported in red

Case N°	MET T	MET M	4EBP1 T	4EBP1 M	S6RP T	S6RP M	mTOR T	mTOR M
1	+	+	+	+	+	+	+	+
2	-	+	+	+	-	+	+	+
3	+	+	+	+	-	-	-	-
4	+	+	+	+	-	-	+	+
5	+	+	+	+	-	+	-	-
6	+	+	+	+	+	+	+	+
7	+	+	+	+	-	-	+	+
8	+	+	-	+	-	+	+	+
9	+	+	+	+	-	+	+	-
10	+	+	+	+	-	+	-	+
11	+	+	+	+	-	+	+	+
12	+	+	-	+	-	+	+	-
13	-	-	-	+	-	+	+	+
14	+	+	+	+	-	+	+	+
15	+	+	+	+	-	+	+	+
16	+	+	+	+	-	+	+	+
17	+	+	+	+	+	+	+	-
18	+	+	+	+	-	+	+	+

19	+	+	+	+	+	-	+	+
20	+	+	+	+	+	+	+	+
21	+	+	+	+	-	-	+	+
22	+	+	+	+	-	-	-	+
23	+	+	+	+	-	+	-	-
24	+	+	+	+	+	+	+	+
25	+	+	+	+	-	-	+	+

Table S4. Intratumor heterogeneity (ITH) between quantitative expression of biomarkers belonging to MET and mTOR pathways in the primary tumor (T) and the respective pulmonary metastasis (M). The ITH grade regarding the intensity of protein expression was widely high for all biomarkers and cases. Heterogeneous cases (for at least one parameter) are reported in red. Only one case demonstrated quantitative homogeneity for all biomarkers

Case N°	MET T	MET M	4EBP1 T	4EBP1 M	S6RP T	S6RP M	mTOR T	mTOR M
1	3	3	2	3	2	2	3	1
2	0	1	1	1	0	1	3	1
3	1	2	3	2	0	1	0	0
4	1	2	2	2	0	0	1	1
5	2	3	3	3	0	1	0	0
6	2	2	3	1	1	3	3	3
7	2	3	3	3	0	NE	3	2
8	2	3	0	3	0	3	3	3
9	1	3	1	3	0	3	2	0
10	1	2	1	3	0	2	0	1
11	1	2	1	3	0	2	3	2

12	1	3	0	3	0	1	3	0
13	0	0	0	2	0	1	2	1
14	3	3	2	3	0	3	2	3
15	1	1	3	3	0	1	3	1
16	1	2	2	3	0	2	2	1
17	1	3	1	3	1	1	3	0
18	1	3	2	3	0	2	2	3
19	2	1	1	2	2	0	3	1
20	2	3	2	3	2	3	2	1
21	2	3	1	3	0	0	1	1
22	3	3	3	3	0	0	0	1
23	3	3	3	3	0	3	0	0
24	1	2	3	2	1	1	3	1
25	1	3	1	3	0	0	3	3

NE = not evaluable
